# Quantification of the NA dependent change of shape in the image formation of a z‐polarized fluorescent molecule using vectorial diffraction simulations

**DOI:** 10.1002/jemt.24060

**Published:** 2022-01-19

**Authors:** Florian Ströhl, Ezra Bruggeman, Christopher J. Rowlands, Deanna L. Wolfson, Balpreet S. Ahluwalia

**Affiliations:** ^1^ Department of Physics and Technology UiT The Arctic University of Norway Tromsø Norway; ^2^ Yusuf Hamied Department of Chemistry University of Cambridge Cambridge UK; ^3^ Faculty of Engineering, Department of Bioengineering Imperial College London London UK; ^4^ Department of Clinical Science, Intervention and Technology Karolinska Institute Stockholm Sweden

**Keywords:** image formation theory, Jones matrix calculus, microscopy, vectorial diffraction

## Abstract

The point spread function of a fixed fluorophore with its dipole axis colinear to the optical axis appears donut‐shaped when seen through a microscope, and its light distribution in the pupil plane is radially polarized. Yet other techniques, such as photolithography, report that this same light distribution in the pupil plane appears as a solid spot. How can this same distribution lead to a spot in one case but a donut in the other? Here, we show how the tube lens of the system plays a critical role in determining this shape. Using a vectorial treatment of image formation, we simulate the relative contributions of both longitudinal and radial components to the image of a dipole emitter and thus show how the donut (typically reported for z‐polarized single molecule fluorescence microscopy) transforms into a solid spot (as commonly reported for photolithography) as the numerical aperture of the tube lens increases. We find that the transition point occurs around 0.7 NA, which is significantly higher than used for most microscopy systems and lower than for common photolithography systems, thus resolving the seeming paradox of dipole shape.

## Graphical abstract

Vectorial diffraction simulations show that the point spread function of a z‐polarized emitter changes shape above a 0.7 NA of the image‐forming lens and can thus impact novel microscopy techniques like oblique plane microscopy in single molecule studies.

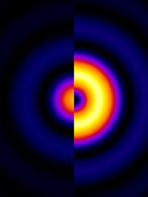

## 1. INTRODUCTION

As a standard, optics textbooks introduce the point spread function of a microscope as the spot‐like Airy disk (Hecht & Zajac, 2002). However, a point source emitter can actually be imaged as many different shapes, the properties of which have been increasingly exploited, particularly for advanced microscopy. For single molecule localization imaging, an emitter's position along the optical axis can be encoded into its shape through the use of a phase mask (e.g., double helix [Pavani et al., 2009] or tetrapod phase masks [Shechtman et al., 2015]) or an asymmetric lens (Huang et al., 2008). Less well‐known is that alternative shapes of single emitters are also possible in microscopes without the use of phase masks or asymmetric lenses; in fact, a simple fixed fluorophore can even appear donut‐shaped. Fluorophores are the unit emitters in fluorescence microscopy and their emission can be modeled as the far‐field of an oscillating linear electric dipole (Novotny et al., 2007). If the dipole axis of such a fluorophore is aligned with the optical axis, the field observed in the image plane of a microscope is a donut (Dickson et al., 1998), while in the pupil plane its emitted electric field is radially polarized. Because this distinct donut shape is lost or deformed as the dipole and optical axis lose alignment, several studies have exploited this property for determining molecular orientation (Beausang et al., 2013; Böhmer & Enderlein, 2003; Forkey et al., 2003; Lippert et al., 2017) or improving superresolution microscopy (Backlund et al., 2012; Mortensen et al., 2010). Intriguingly, this very same *radially polarized* pupil plane distribution of light is used in, for example, photolithography (Quabis et al., 2000; Zhan, 2009) with a completely different outcome. There, a radially polarized vector beam illuminates the pupil plane, creating a field equivalent to that of the dipole emitter (but typically of higher intensity due to application needs), which in turn generates a very tight focal spot in the sample plane. These sharp spots are important for applications as diverse as high‐resolution lithography (Lin et al., 2015; Shoham et al., 2006), optical data storage (Zhan, 2009), laser cutting (Niziev & Nesterov, 1999), or for trapping and manipulating nanoscale particles (Zhan, 2009).

**FIGURE 1:**
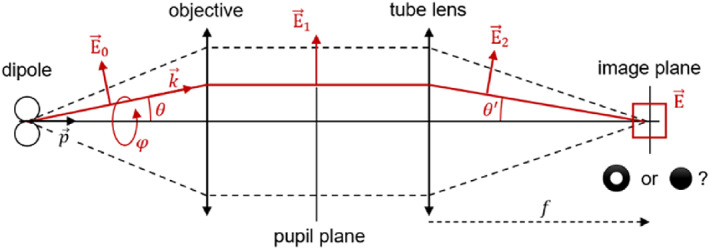
Optical planes of a microscope. The electric far‐field emitted by a dipole is captured by the objective lens and refracted to the pupil plane. The field distribution is, for the case depicted here of a dipole aligned co‐linear to the optical axis, purely radially polarized; this distribution is akin to the field distribution of a vector beam. In the image plane, two alternatives are commonly seen: (1) the point emitter is imaged with a dip in the intensity at the Centre (a “donut”) as known from single molecule microscopy, or (2) the emitter appears as a tight spot, as used in applications such as direct laser writing with radially polarized beams in photolithography, machining, or optical data storage

This raises the question: if the fields are equivalent at the pupil plane, why are they different at the image plane? The obvious answer is that there must be something different between the pupil and imaging plane in those two situations, and looking at Figure 1, the culprit appears to be the tube lens (or the “image‐forming” lens for non‐microscopy applications). Indeed, a closer look at the setups used for microscopy versus those for, for example, photolithography reveals that the NAs of their image‐forming lenses are strikingly different. Commonly used tube lenses have NAs well below 0.1, whereas the focusing lenses in lithography systems have NAs closer to 1. What may not be obvious, however, is why the difference in NA has such a significant impact on the image shape. In the following, we first qualitatively explore the basis for this difference in shape, and then use vectorial diffraction simulations to quantitatively investigate the transition from one form to another as the NA of the tube lens is gradually increased.

**FIGURE 2:**
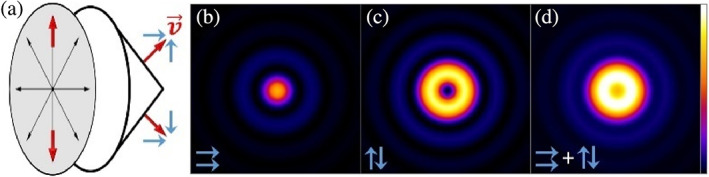
Longitudinal and radial field contributions to dipole image formation. (a) The field in the pupil plane (gray disk) is radially polarized. Two points are highlighted as red arrows to exemplify the pair‐wise anti‐parallel polarization vectors. After refraction by the lens, the polarization vector $\vec{v}$ of the corresponding plane waves can be split into polarization components in longitudinal and radial directions (blue arrows). (b) The longitudinal components are parallel and in phase with each other and thus interfere constructively into a tight spot, whereas (c) the anti‐parallel radial components cancel each other out in the image center due to destructive interference. (d) The actual observed distribution is formed by summation of both components. The plots in (b–d) show the normalized intensity from simulated imaging of a dipole using a 0.95 NA objective together with a 0.55 NA tube lens

An intuitive explanation for this phenomenon may be found by splitting up points in the pupil plane into pairs. Let us have a closer look at the relevant part of the microscope as depicted in Figure 2a, encompassing the pupil plane and the image‐forming lens. Each pair of points in the pupil plane is related by inversion symmetry through the center of the pupil. The red arrows visualize the electric field directions of one such point pair. As the field in the pupil is radially polarized, the arrows point in opposite directions. Equivalently, one could state that the points share the same linear polarization vector but with an effective relative phase shift of π between them ($e^{i\pi }=-1$).

After refraction by the lens, any point on the pupil will produce a corresponding plane wave, and the polarization vector $\vec{v}$ of this plane wave can be divided into a longitudinal component $v_{l}$ and a radial component $v_{r}$ with the relative weighting dependent on the angle between the propagation direction and the optical axis. Geometrically, one can see that the point pair components in the longitudinal direction (⇉) result in constructive interference (Figure 2b), whereas the radial components (↑↓) will interfere destructively and cancel each other out in the image center (Figure 2c). The observed image of a dipole is therefore the sum of these two interference patterns (Figure 2d); in lenses with high numerical aperture the longitudinal components of $\vec{v}$ are dominant and the focus appears as a spot, whereas in low NA lenses (like conventional microscope tube lenses) the radial term dominates, and the “donut” appears. It is hence also absolutely expected to find both cases reported in literature as both the donut and the tight focus are possible manifestations of the same phenomenon.

While several groups have contributed to an increased understanding of image formation in high‐NA vector beam focusing (Brown, 2011; Lan & Tien, 2008; Quabis et al., 2000; Sheppard & Choudhury, 2004; Sheppard & Török, 1997; Youngworth & Brown, 2000), none has yet answered a key question: where *exactly* does the donut turn into a spot? This transition point can be accurately determined by calculating the image of a dipole but will require several steps as discussed below.

## 2. METHODS

First, the electric far field $E_{0}$ emitted by the dipole needs to be expressed, which can be done elegantly (Kim et al., 2018) by concatenation of vector products between a wave vector $\vec{k}\left(\varphi ,\theta \right)$ and the emission dipole moment $\vec{p}={\left[p_{x},p_{y},p_{z}\right]}^{T}$. See Figure 1 for an overview of the used variables. Here, we consider the optical axis to be along the z direction. Expressed in Cartesian coordinates, the wave vector $\vec{k}={\left[\sin\theta \cos\varphi ,\sin\theta \sin\varphi ,\cos\theta \right]}^{T}$ is a function of polar angle φ and azimuthal angle θ, and the field ${\vec{E}}_{0}$ in object space *before* the objective is

(1) $${\vec{E}}_{0}=\left(\vec{k}\times \vec{p}\right)\times \vec{k}.$$

The portion of this field up to $\theta_{\max}$ (the half‐opening angle of the objective), is captured and refracted by the microscope objective. Refraction can be modeled via symbolic ray tracing with vectorial Jones matrices (Kim et al., 2018). Two matrices are important for lens refraction. The first is the rotation matrix $R_{z}$, which changes the Cartesian coordinate system $\left(x,y,z\right)$ to the coordinate system spanned by the meridional and sagittal unit vectors $\left(x_{m},y_{s},z\right)$ of the currently traced ray. The second important matrix, L, models the ray refraction caused by a lens and redirects all meridional vector components. This ray refraction matrix is also a rotation matrix (any ray coming from the focus of the lens is parallel to the optical axis after the lens, which is achieved via rotation of the ray by its azimuthal angle θ):

(2) $$R_{z}=\left[\begin{array}{ccc} \cos\varphi & \sin\varphi & 0\\ -\sin\varphi & \cos\varphi & 0\\ 0 & 0 & 1 \end{array}\right],L=\left[\begin{array}{ccc} \cos\theta & 0 & \sin\theta \\ 0 & 1 & 0\\ -\sin\theta & 0 & \cos\theta \end{array}\right].$$

Note that the ray refraction matrix L also contains an apodization factor of either $A\left(\theta \right)=\sqrt{\frac{n_{2}}{n_{1}}\left|\frac{1}{\cos\left(\theta \right)}\right|}$ in aplanatic collimation, or its inverse $A\left(\theta \right)=\sqrt{\frac{n_{1}}{n_{2}}\left|\cos\left(\theta \right)\right|}$ in aplanatic focussing (Kim et al., 2018) (where $n_{1}$ and $n_{2}$ are the refractive indices before and after the lens). Further, for clockwise ray refraction, the azimuthal angle θ in L needs to be used with a negative sign (Kim et al., 2018). Depending on the type of condition the lens is made to obey (Abbe, Herschel, Lagrange, Helmholtz), different forms of apodization should be used (Gu, 2000); objective lenses for microscopy are designed to obey the Abbe sine condition (Gu, 2000). The pupil plane electric field ${\vec{E}}_{1}$ is thus a multiplication of the object‐space electric field with a rotation matrix, the objective lens refraction matrix $L_{O}$ and a following inverse rotation matrix $R_{z}^{-1}$ to restore the Cartesian $\left(x,y,z\right)$ coordinates:

(3) $${\vec{E}}_{1}=R_{z}^{-1}L_{O}R_{z}{\vec{E}}_{0}.$$

Following these operations, the z‐component of the electric field ${\vec{E}}_{1}$ in the pupil plane vanishes. This is also intuitively correct since the refracted electric field is invariant along the axial direction and thus possesses only electric field components in the $\left(x,y\right)$ plane. All p‐polarized electric field components (i.e., the ones in the meridional plane) before the objective are hence converted into radial components, whereas all s‐polarized electric field components of the dipole far field become azimuthal polarization components. For the special case of a dipole with its axis along the optical axis, the electric field ${\vec{E}}_{1}$ in the pupil plane contains only radial components (${\vec{E}}_{0}$ has no azimuthal components, so neither does ${\vec{E}}_{1}$).

Let us continue with the special case. Seen from the perspective of the objective lens, the dipole's electric field is p‐polarized regardless of emission direction and therefore, after refraction by the objective lens, the electric field in the back focal plane is purely radial. Further, the strength of the dipole emission in the pupil plane is proportional to $\sin\left(\theta \right)$. This is contained within Equation (1): the magnitude of the cross product $\vec{k}\times \vec{p}$ is the sine of the angle between the two vectors and in our special case, the dipole moment is along the optical axis, that is, $\vec{p}={\left[0,0,1\right]}^{T}.$ As the consecutive cross product with k is a cross product with an orthogonal unit vector, it does not change the magnitude. Further, for a dipole with its moment $\vec{p}$ on the optical axis the angle enclosed with wavevector $\vec{k}$ is just the angle θ of the spherical coordinate system. Therefore, the amplitude distribution in the Fourier plane is $\sin\left(\theta \right)$ times an additional apodization factor $A\left(\theta \right)$ due to aplanatic collimation of the objective lens. Taken together, the pupil plane electric field is radially polarized and has a vanishing field strength in the pupil center. If, as implicitly assumed, the dipole is placed in the focus of the objective on the optical axis, the electric field's phase in the pupil plane is constant over the entire pupil and thus is equivalent to a radially polarized vector beam.

Calculation of the image of such a dipole is sometimes simplified in single‐molecule microscopy as the image‐forming tube lens can be regarded as a paraxial lens (Backer & Moerner, 2014) (an example for a fully vectorial treatment of the imaging system is given in Böhmer and Enderlein (2003). Under the paraxial approximation, it is sufficient to take the Fourier transform of the dipole electric field distribution in the pupil plane. As the amplitude distribution in the pupil is an even function, while the direction of the electric field is equivalent to an odd phase function, this makes the overall pupil function odd. A property of the Fourier transform is that odd functions remain odd, so there must be a zero‐crossing at the image plane origin. Addition of the squares of the resulting electric fields in the x and y directions yields then the overall intensity distribution in the shape of a donut (Böhmer & Enderlein, 2003).

In contrast, the tight spot in vector beam focusing is the result of the omitted non‐paraxial part and hence requires use of a non‐paraxial diffraction integral. The Debye integral is such an integral and synthesizes the field distribution $\vec{E}$ in the focal region from a plane wave spectrum ${\vec{E}}_{2}$ (Leutenegger et al., 2006). Importantly, it requires the plane wave spectrum ${\vec{E}}_{2}$ *after* the tube lens, so we need to propagate the field in the pupil once more:

(4) $${\vec{E}}_{2}=R_{z}^{-1}L_{\mathrm{TL}}R_{z}{\vec{E}}_{1}$$

The tube lens refraction matrix $L_{\mathrm{TL}}$ now incorporates the apodization factor for focusing (i.e., the inverse of Equation (2)) and θ in $L_{\mathrm{TL}}$ is the azimuthal angle *in image space* $\theta^{\prime }$. As both lenses, objective and tube lens, can be assumed to obey the Abbe sine condition, we can link the azimuthal angles of object space and image space, θ and $\theta^{\prime }$, through the system magnification $M=\frac{n\sin\left(\theta \right)}{n\prime \sin\left(\theta^{\prime }\right)}.$ Here, the refractive index of the immersion medium in object space is n, while the refractive index in image space is n′=1. For air objectives, the refractive indices can be omitted. The Debye integral is now performed over the plane wave spectrum in image space $\left(\theta \prime \right)$:

(5) $$\vec{E}\left(x,y,z\right)=-\frac{\text{if}}{\lambda_{0}}\int_{0}^{\theta_{\max}^{\prime }}\int_{0}^{2\pi }\sin\left(\theta \prime \right){\vec{E}}_{2}\left(\theta \prime ,\varphi \right)e^{i\left(k_{z}z-k_{x}x-k_{y}y\right)}\;\mathrm{d\varphi}\;\mathrm{d\theta}\prime$$

Here, i is the imaginary unit and the pre‐factor uses the focal length f of the lens and the wavelength $\lambda_{0}$. The half‐opening angle of the image‐forming lens, $\theta_{\max}^{\prime },$ is related to the numerical aperture of the objective via $n\prime \sin\left(\theta_{\max}^{\prime }\right)=NA_{O}/M$. Following the approach by Leutenegger et al. (2006), a significantly faster computation of the integral in Equation (4) can be obtained by changing the integration variables through a coordinate transform from $\left(\varphi ,\theta \prime \right)$ to $\left(k_{x},k_{y}\right)$ to be over the support region of the pupil plane (Leutenegger et al., 2006):

(6) $$\vec{E}\left(x,y,z\right)=-\frac{\text{if}}{\lambda_{0}k\prime^{2}}F\left\{\frac{{\vec{E}}_{I}\left(\theta \prime ,\varphi \right)e^{ik_{z}z}}{\cos\left(\theta \prime \right)}\right\}.$$

The factor $k\prime =2\pi n\prime /\lambda_{0}$ is the wavevector magnitude in image space. The final image intensity I is obtained by multiplication of the electric field in image space E with its conjugate transpose, that is, $I=EE^{†}$.

## 3. RESULTS

**FIGURE 3:**
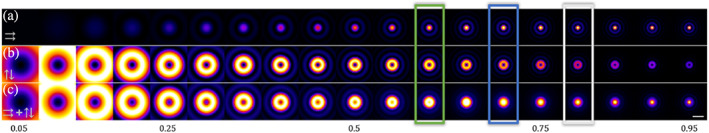
The image of a dipole emitter (colinear to the optical axis), recorded by an objective lens of 0.95 NA, as formed by a tube lens with NAs in the range from 0.05 to 0.95. Shown are the intensities produced by (a) the longitudinal, (b) radial, and (c) all field components. Intensities are normalized separately for each tube lens NA. The colored boxes highlight “turning points”: At ~0.6 NA (green box), the maximum intensity of the longitudinal and radial components are equal; at ~0.7 NA (blue box), the central intensity of the total intensity has a stationary point; and at ~0.8 NA (white box), the total energy contributions of the longitudinal and radial components are equal. The scale bar is 1μm and the wavelength used in the simulation was 500nm

The resulting intensity distribution for a range of tube lens numerical apertures is visualized in Figure 3. There, the pixel size is 100nm over a field of view spanning 25μm in all spatial dimensions and the simulated wavelength was 500nm. Note how the relative sizes of the donut or spot change with the ratio of the numerical aperture between objective and tube lens. The light‐capturing lens $L_{O}$ in Figure 3 was modeled in all cases with 0.95 numerical aperture and thus did not influence the formed dipole images. Clearly, the controlling factor between the donut and the spot is the ratio of the longitudinal and radial field contributions, which are balanced by the image‐forming lens. Yet to answer the question about where exactly the turning point between the donut and the spot lies, one needs to first define “turning point.” Three answers can easily be given, as indicated by the colored boxes in Figure 3. One could equate the (1) maximal intensity values of radial and longitudinal images or, similarly, (2) their total energy contribution. As an alternative, (3) a zero‐crossing of the second derivative of the total image intensity on the optical axis can be taken. In the latter, note that the direction is arbitrary due to symmetry. The turning point in this case would be equal to a stationary point of the intensity distribution.

The ratio of maxima yields a turning point at a tube lens NA just above 0.6, which agrees with reported results in cylindrical vector beam focusing (Biss et al., 2003). In the case of dipole imaging, however, the overall intensity distribution still possesses a local minimum, as the peripheral region of the “spot” adds incoherently to the donut, and thus does not inform about our sought‐after donut‐to‐spot turning point. Taking the ratio of total intensity contributions results in a turning point around 0.8 NA. Interestingly, this definition makes the “turning point” more dependent on the objective NA. At a higher objective NA, the periphery of the pupil receives a relatively higher field strength compared to the pupil center, which contributes mostly to the longitudinal “spot” portion of the overall image. We see that this effect is present also in the other metrics, albeit to a lesser extent. Using a sign change of the local curvature of the image center as a metric (i.e., when the center no longer has a donut “hole”) yields a turning point near a tube lens NA of 0.7, which is in‐between the other two metrics. While this definition is closest to an intuitive understanding of “turning point” between a donut and a spot, it lacks, however, the direct connection to the field components that the other two definitions maintain.

**FIGURE 4:**
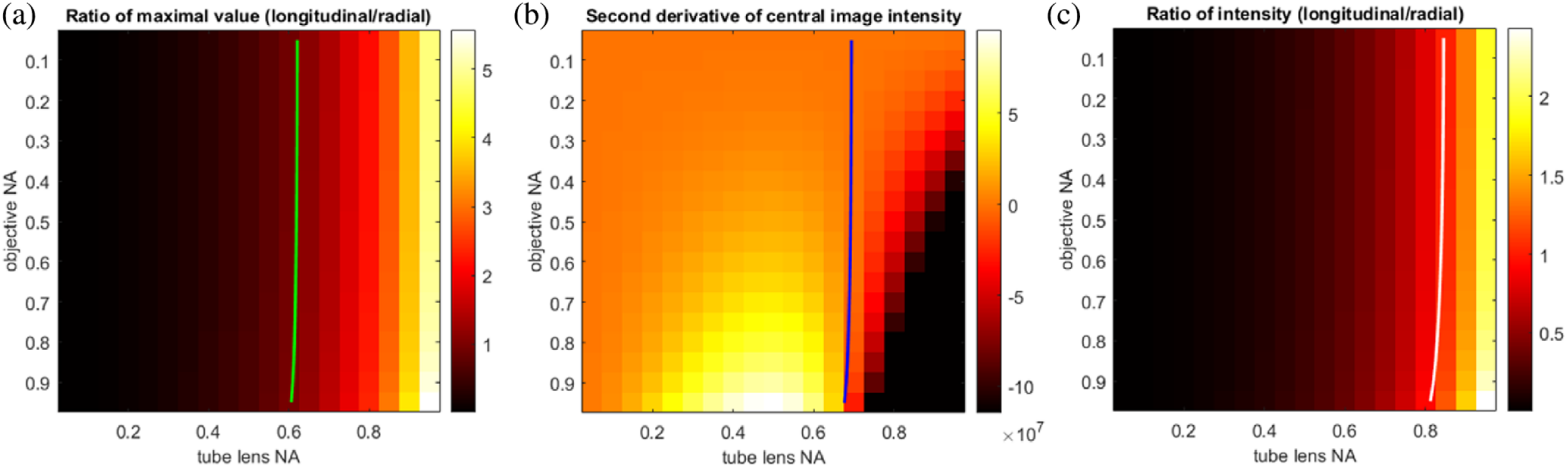
The “turning point” between donut and spot‐like images can be defined in various ways: (a) equality between the maximum intensity of longitudinal and radial components, (b) stationary point of the image center, or (c) equality of total energy contributions of longitudinal and radial components. Plotting the turning point for a range of objective NAs results in a “turning line”; the color of these lines matches the colors of the corresponding turning point boxes in Figure 3. Higher objective NAs result in equality of longitudinal and radial field components at smaller tube lens NAs. This is caused by the higher field strength at the periphery of the pupil when higher NA objective lenses are used. Note that in (b) negative values are saturated to better visualize the turning line

Thus far the “turning point” has been defined as the NA of the tube lens where the donut changes to a spot, but as shown in Figure 4 it also depends to a smaller degree on the NA of the objective lens; adding this dimension, our “turning point” becomes a “turning line.” This small effect is due to the azimuthal field strength dependence of dipole emission. With higher NA, the amplitude distribution in the Fourier plane becomes steeper when moving from the middle to the periphery, and consequently more energy is funneled into the longitudinal field component. Note that immersion objectives with numerical apertures >1, in particular those that can capture evanescent field components, are not included in the present analysis. From this analysis, we can deduce that—as long as the back‐aperture of the tube lens is filled—the magnification M of the whole system does not play a significant role, even though M can be orders of magnitude different between high‐resolution microscopy and lithography.

## 4. DISCUSSION AND CONCLUSION

Conventional microscopes operate with tube lens NAs below 0.1, so z‐polarized dipoles are always imaged as donuts. In single molecule localization microscopes, this donut shape can negatively impact the achievable localization precision due to algorithmic assumptions of standard, point‐like light distributions. So far, this effect has been counteracted by modifying the pupil plane (Aguet et al., 2009; Lewog & Moerner, 2014). In general, pupil‐plane polarization and phase masks can be utilized to enable constructive interference (as opposed to destructive) at the central image point; this has been successfully applied in the contexts of z‐polarized confocal imaging (Huse et al., 2001) and single‐molecule (widefield) microscopy (Lew & Moerner, 2014). Both approaches, however, require access to the pupil plane, which in turn requires additional optical relays that cause a lower overall transmission efficiency of the system. As detectors' pixel sizes continue to shrink (the latest smart phone cameras reach pixel sizes as small as 600nm), it might become feasible to use higher NA tube lenses to both achieve Nyquist sampling and simultaneously exploit the intrinsic spot shape due to the dipole's longitudinal field. Care must be taken, however, as the image is sensed not in air, but inside a (typically silicon) sensor, whose index of refraction is about 3.5 and usually has dielectric coatings. In previous analyses on cylindrical vector beam focusing (Biss et al., 2003), it was shown that strong refraction at a silicon surface severely diminishes the longitudinal field component inside the high‐index material, leaving mostly the lateral component, and thus the donut shape, behind. A *pixelated* display, in contrast to a continuous silicon slab, would impose different boundary conditions on the field formation though, which would further modify this result. As such an analysis requires a finite element method for simulation of the focal region, it cannot be answered here using our approach.

Outside of single molecule localization microscopy, another field where this donut transition, as well as the above‐mentioned camera miniaturization, can have significant future impacts is neuroscience. Here, studies are often conducted by strapping tiny microscopes to the heads of animals during stimulation experiments; the equipment therefore must have minimal size and weight so as to not perturb the animals too much. A smaller imaging chip also comes with the possibility of using a higher NA GRIN (GRadient‐INdex) lens for imaging; here a single lens serves as both objective and image forming lens. For instance, the recently published “miniscope 3D” (Yanny et al., 2020) uses a GRIN lens with 0.55 NA as the sole optical element. This is notably close to the turning point of a donut as shown above and could have strong implications for the interpretation of imaging results. Future applications of this technology may therefore be well‐advised to take vectorial effects into stronger consideration.

Further, with the advent of various microscopy techniques that use high numerical aperture lenses in their light path “downstream” of the detection objective apart from the tube lens, awareness of effects due to the vectorial character of light is becoming much more important in microscopy. For example, the single objective light sheet technique, oblique plane microscopy (OPM), (Dunsby, 2008) actually uses two additional objectives in its light path. The secondary objective is used to construct a so‐called “perfect imaging” relay to observe an aberration‐free tilted image plane using a tertiary objective. The latest OPM techniques employ a refractive index boundary between the secondary and tertiary objectives (Yang et al., 2019) to increase the overall collection efficiency, but this leads to asymmetric Fresnel transmission coefficients of the refractive index boundary. These are expected to lead to different strengths of the image‐forming ray components depending on their polarization and can thus impact image formation.

Taken together, our results provide valuable knowledge of vectorial point spread function shapes in high NA situations, which are becoming increasingly relevant in latest microscopy systems.

## CONFLICT OF INTEREST

The authors declare that there are no conflicts of interest.

## Data Availability

The data/software that support the findings of this study are openly available in DataverseNO at https://doi.org/10.18710/Z37SBD, reference number Z37SBD.
